# Supplementary material for: Arabidopsis in the Wild—The Effect of Seasons on Seed Performance
Source: Plants (Basel). 2020 May 1;9(5):576. doi: 10.3390/plants9050576 (PMC7285089; doi:10.3390/plants9050576)
Supplement: Supplementary file 1 [file plants-09-00576-s001.pdf]

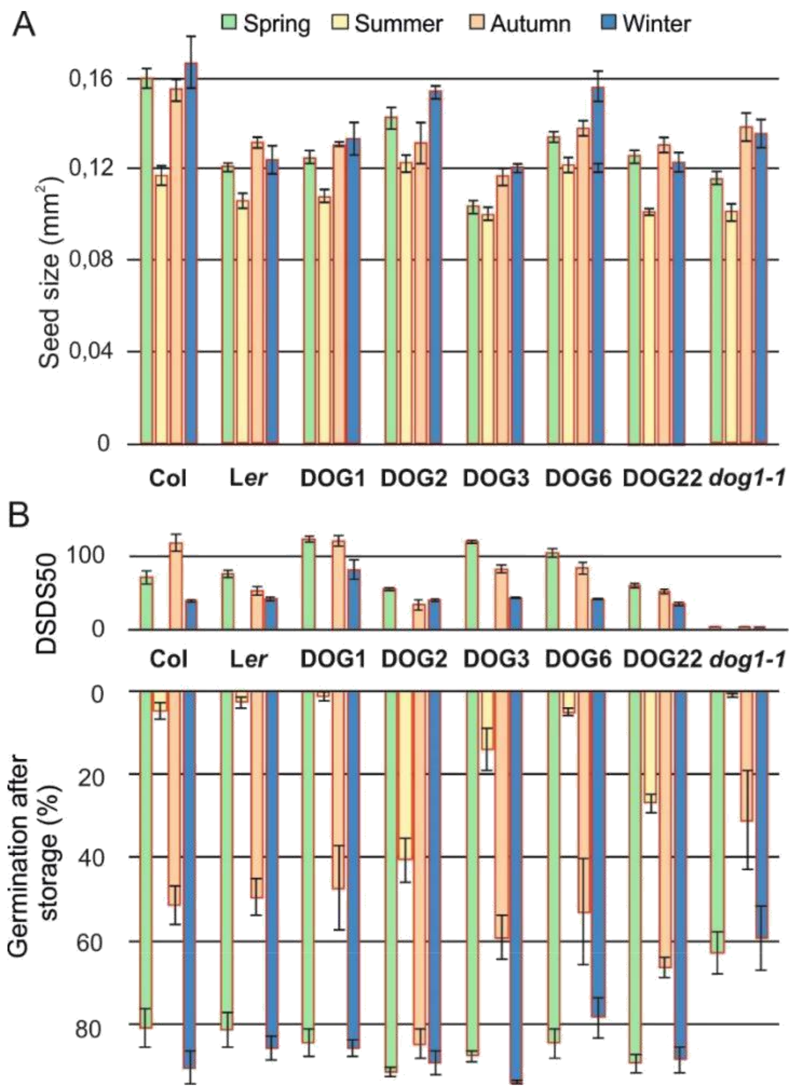

Figure S1. Seed performance of the individual genotypes over four season. (**A**) Seed size (mm^2), (**B**) Seed Dormancy (DSDS50) and seed germination after storage (longevity) levels for each genotype for all seasons of 2014. Averages of four replicates are displayed. Error bars represent standard errors.

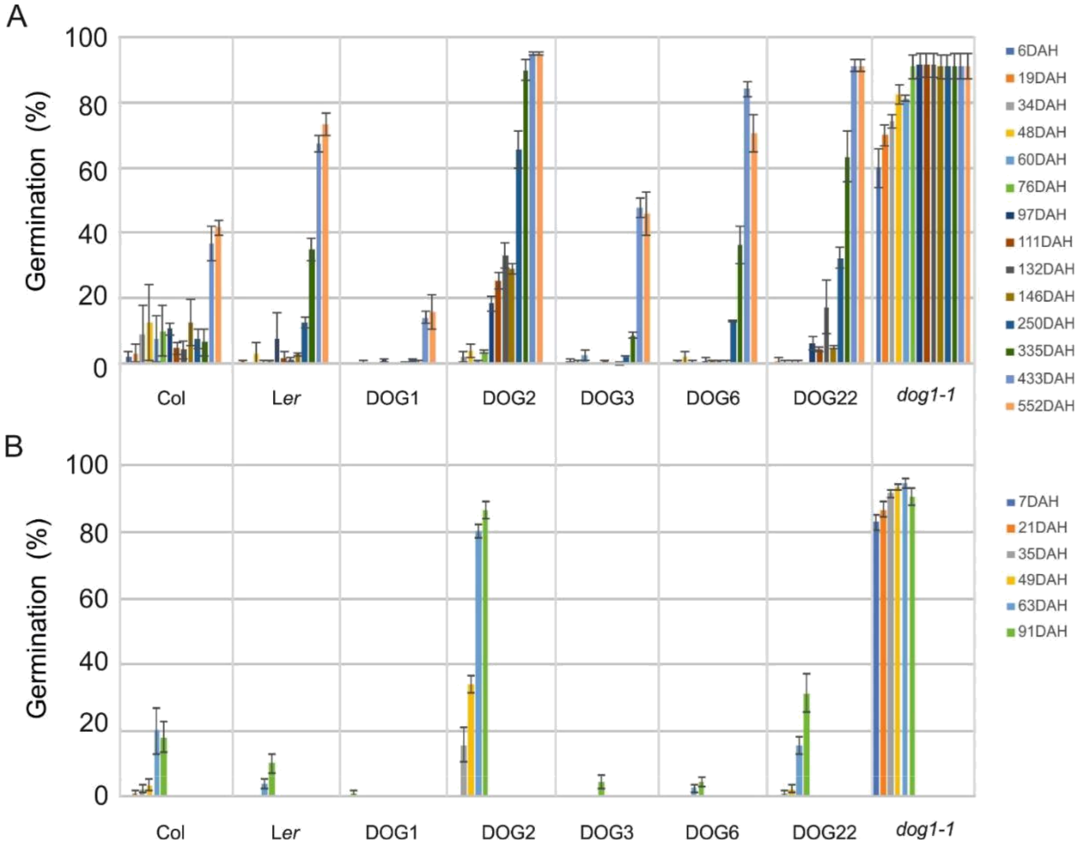

Figure S2. After-ripening requirement for seeds of the summer experiments. (**A**) Seeds produced in 2012. Release of dormancy was followed until 552 Days After Harvest (DAH) at which most lines reach over 50% of germination. (**B**) Seed produced in 2014. Release of dormancy was followed until 91 DAH. Averages of four replicates are displayed. Error bars represent standard errors.

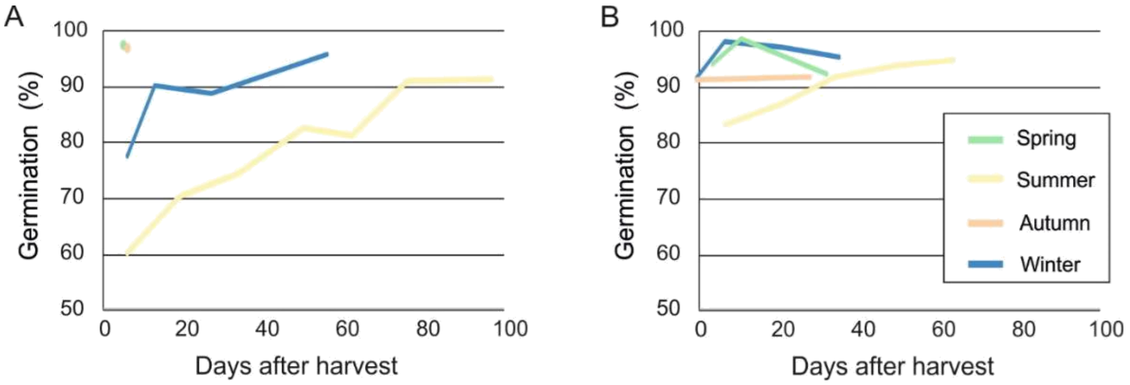

Figure S3. After-ripening requirement for *dog1-1* seeds produced in all seasons of 2012 and 2014. (**A**) Seeds produced in 2012. (**B**) Seed produced in 2014. Release of dormancy was followed until 91 DAH. Release of dormancy was followed until seeds reached over 90% germination. Averages of four replicates are displayed.
